# Stress-induced epinephrine promotes hepatocellular carcinoma progression via the USP10-PLAGL2 signaling loop

**DOI:** 10.1038/s12276-024-01223-0

**Published:** 2024-05-01

**Authors:** Chen Wang, Jiaping Ni, Dongqing Zhai, Yanchao Xu, Zijie Wu, Yuyuan Chen, Ning Liu, Juan Du, Yumeng Shen, Guilai Liu, Yong Yang, Linjun You, Weiwei Hu

**Affiliations:** 1 Center for New Drug Safety Evaluation and Research, State Key Laboratory of Natural Medicines, China Pharmaceutical University, Nanjing, 211198 PR China; 2 Department of Hepatobiliary Surgery, Nanjing Drum Tower Hospital Clinical College of Jiangsu University, Nanjing, PR China; 3 Department of Pharmacology, College of Pharmacy, Ningxia Medical University, Yinchuan, 750001 Ningxia PR China; 4 Lingang Laboratory, Shanghai, 200032 PR China

**Keywords:** Liver cancer, Epithelial-mesenchymal transition

## Abstract

Hepatocellular carcinoma (HCC) is associated with a poor prognosis. Our previous study demonstrated that Pleomorphic adenoma gene like-2 (PLAGL2) was a potential therapeutic target in HCC. However, the mechanisms that lead to the upregulation of PLAGL2 in HCC remain unclear. The present study revealed that stress-induced epinephrine increased the expression of PLAGL2, thereby promoting the progression of HCC. Furthermore, PLAGL2 knockdown inhibited epinephrine-induced HCC development. Mechanistically, epinephrine upregulated ubiquitin-specific protease 10 (USP10) to stabilize PLAGL2 via the adrenergic β-receptor-2-c-Myc (ADRB2-c-Myc) axis. Furthermore, PLAGL2 acted as a transcriptional regulator of USP10, forming a signaling loop. Taken together, these results reveal that stress-induced epinephrine activates the PLAGL2-USP10 signaling loop to enhance HCC progression. Furthermore, PLAGL2 plays a crucial role in psychological stress-mediated promotion of HCC progression.

## Stress Hormone Boosts Liver Cancer Progression via PLAGL2

Hepatocellular carcinoma (HCC, a type of liver cancer) is a major cause of cancer deaths globally, with high chances of recurrence and spread. Scientists discovered that the protein PLAGL2 contributes to HCC’s growth and spread, but it’s unclear how its levels increase. Weiwei Hu and his team aimed to explore this. They performed various tests using HCC tissues, cell cultures, and mice. They discovered that long-term stress, specifically the hormone epinephrine, boosts the production of PLAGL2 and HCC progression. This study offers new understanding of stress’s role in cancer progression and potential targets for HCC treatment. The scientists concluded that more research is required to fully comprehend these results for future cancer therapies.

This summary was initially drafted using artificial intelligence, then revised and fact-checked by the author.

## Introduction

Hepatocellular carcinoma (HCC) is one of the leading causes of cancer-associated mortality worldwide^1^. Despite the emergence of novel therapies such as immune checkpoint inhibitors, HCC is associated with a poor prognosis and high recurrence and metastasis rates. Our previous study revealed that the transcription factor PLAGL2 was involved in the proliferation and metastasis of HCC^2^. However, the molecular mechanism underlying the upregulation of PLAGL2 expression has not been determined. Therefore, the need to investigate the mechanism underlying the upregulation of PLAGL2 is urgent.

PLAGL2 is a zinc-finger transcription factor in the PLAGL gene family^3^. Previous studies have reported that PLAGL2 is involved in the progression and metastasis of multiple cancers. Wu et al. reported that PLAGL2 promoted the proliferation and migration of gastric cancer cells through ubiquitin-specific protease 37 (USP37)-mediated deubiquitylation of the Snail1 protein^4^. Furthermore, studies have demonstrated that PLAGL2 is involved in actin cytoskeleton structure modulation and cell migration^5^. PLAGL2 enhances cell proliferation by inducing the G1 to S transition. In addition, PLAGL2 cooperates with the leukemia fusion protein Cbfb-SMMHC in the development of acute myeloid leukemia (AML)^6^. A previous study showed that PLAGL2 interacted with RING-H2 protein (Pirh2), a p53-inducible E3 ligase involved in the ubiquitination of p53. A study revealed that PLAGL2 was an oncoprotein that negatively regulated the levels and stability of p53^7^. Hu et al. showed that PLAGL2 knockdown attenuated the proliferation and metastasis of HCC cells by decreasing signaling through the PI3K-Akt pathway. Moreover, PLAGL2 acted as a transcription regulator of EGFR and decreased sensitivity to erlotinib^2^. These findings indicate that abnormal PLAGL2 expression plays a key role in tumor development.

Recently, we found that PLAGL2 upregulation was associated with chronic stress. Increasing evidence shows that depression is associated with poorer prognosis in cancer patients. Indeed, chronic stress activates the sympathetic nervous system (SNS) and the hypothalamic‒pituitary‒adrenal (HPA) axis, leading to increased catecholamine and cortisol hormone secretion. Catecholamines activate signaling pathways, such as the PI3K-AKT-mTOR, IL-6-STAT3, and Hippo-Yap pathways, and upregulate proto-oncogenes, such as Erk, Src, and c-Myc^8^. Recent evidence shows that chronic stress influences 9 of the 14 hallmarks of cancer defined in 2022^9–11^. Furthermore, several previous studies revealed that chronic stress was associated with recurrence and metastasis in HCC^12,13^. Lin X reported that stress-induced norepinephrine stimulated hepatic stellate cells (HSCs) to secrete secreted frizzled-related protein 1(sFRP1) to promote HCC progression via the Wnt16B/beta-catenin pathway^14^. Liu J et al. demonstrated that chronic stress mediated HCC metastasis through the β2-AR/YB-1/β-catenin pathway^15^. However, the molecular mechanism linking chronic stress and PLAGL2 has not been determined.

Here, we identified the role of stress-induced epinephrine and identified a mechanism underlying the upregulation of PLAGL2. Epinephrine could promote PLAGL2 expression and HCC progression. A functional study showed that epinephrine increased the metastasis of HCC cells in vitro and in vivo. A mechanistic study showed that epinephrine activated the PLAGL2-USP10 signaling loop via the ADRB2-c-Myc axis. Our findings primarily demonstrated a novel mechanism linking psychological stress with PLAGL2 upregulation and HCC progression, suggesting the therapeutic efficacy of targeting PLAGL2.

## Materials and methods

### Hepatocellular carcinoma (HCC) tissue samples

HCC tissue samples were surgically resected from patients at Nanjing Drum Tower Hospital (Jiangsu, China). Fresh surgical specimens were immediately frozen in liquid nitrogen and stored at −80 °C for analysis. An ethics permit was obtained from the Ethics Committee of the local hospital, and informed consent was obtained from all patients involved in the study (Approval no. 82073280). The information on the human tissue samples is summarized in Supplementary Table 5.

### Cell lines and culture conditions

The human HCC cell lines (Huh-7, HCCLM3, and Hep3B) and the murine HCC cell lines Hepa1-6 and 293T cell were purchased from the Chinese Academy of Science Shanghai Cell Bank. Huh-7, HCCLM3, and 293T cells were maintained in Dulbecco’s modified Eagle’s medium (DMEM) supplemented with 10% FBS and 1% penicillin‒streptomycin (PS). Hep3B cells were maintained in Eagle’s minimum essential medium (MEM) supplemented with 10% FBS and 1% PS. The cells were incubated in a humidified incubator containing 5% CO_2_ at 37 °C.

### Chronic stress model and xenograft mouse model

C57BL/6N male mice (4–6 weeks) were subjected to chronic stress by being restrained in 50 mL centrifuge tubes punctured with small holes to allow them to breathe. The mice were stressed for 6–8 h per day for 14 days. On the 14^th^ day, the mice were subcutaneously injected with 0.1 ml of Hepa1-6 cells (2 × 10^6^ in PBS/Matrigel [1:1]). The mice were then maintained under stressed conditions for two additional days. Successful generation of the model was determined by depression-like behavior tests and measuring serum levels of catecholamines.

### Animal studies

Four- to six-week-old C57BL/6N mice and nude mice were randomly allocated to the epinephrine-treated, PBS-treated, or propranolol-treated groups. The epinephrine-treated group was subcutaneously injected with epinephrine (6 mg/kg/d), while the propranolol-treated group received an intraperitoneal injection of propranolol (2 mg/kg/d). Both groups were treated with the drugs for 14 days before being subcutaneously injected with Hepa1-6 cells (for C57BL/6N mice), H22 cells (for BALB/c mice), or HCCLM3 cells (for BALB/c-nu mice) (2 × 10^6^ cells were dissolved in 0.1 ml of PBS/Matrigel [1:1]). Furthermore, a metastatic mouse model was established by injecting 4- to 6-week-old male NOD/SCID mice with HCCLM3 shPLAGL2 or HCCLM3 shCtrl cells (1 × 10^6^ in 0.05 ml of PBS) into the spleen envelope following 14 days of epinephrine treatment. Six weeks after tumor implantation, the mice were subjected to in vivo scans before being sacrificed. The DEN+CCl4-induced HCC model was established by intraperitoneally injecting 14-day-old male PLAGL2^f/f^ Alb-cre and PLAGL2^f/f^ C56BL/6N mice with diethylnitrosamine (DEN, 20 mg/kg), after which the mice were randomly assigned to the epinephrine-treated group or the control group. From week four of age until the end of the experiment, the mice received once-weekly intraperitoneal injections of CCl4 (1 ml/kg dissolved in soybean oil).

### Plasmids, siRNA, and cell transfection

Plasmids encoding full-length human USP10, the USP10 promoter, and USP10 mutant plasmids were purchased from You Bao Biotechnology Company. USP10 and c-Myc siRNAs were purchased from GenePharma. The plasmids and siRNAs were transfected with jetPRIME transfection reagent (Polyplus-Transfection) at a final concentration of 50 nmol/L according to the manufacturer’s instructions.

### Wound-healing and cell migration assays

For the wound-healing assay, HCC cells (3 × 10^5^) were seeded in six-well plates and allowed to form a confluent monolayer. Upon reaching confluence, the cells were horizontally scratched with pipette tips and incubated in a serum-free medium. For each well, at least three images were taken at the indicated time points using a Leica DMI 3000 B microscope. The percentage of wound healing was determined based on at least three measurements of the wound area. For the migration assay, HCC cells (1 × 10^4^) were seeded in the upper chamber (12-well insert, Corning Costar, China) with serum-free medium and incubated for 36 h. In the bottom chamber, a medium supplemented with 10% FBS was added to act as an attractant. Before being imaged, the cells in the chambers were fixed with 4% paraformaldehyde (Santa Cruz) and stained with crystal violet (Shanghai Sangon Company, China). Images were captured using a Leika DMI 3000 B microscope.

### Quantitative reverse transcriptase-PCR

Total RNA was extracted using TRIzol reagent (TAKARA, AA7002). HiScript III RT SuperMix for qPCR with gDNA eraser (R323, Vazyme) and oligo-dT primers were used to generate cDNA. Then, real-time reverse transcriptase-PCR was performed using ChamQ SYBR qPCR Master Mix (Q331, Vazyme) according to the manufacturer’s instructions. *ATCB* was used as an internal control. The primers used in the experiments are listed in Supplementary Tables 1–4.

### Western blot analysis

The cells were lysed in RIPA/IP buffer on ice. Then, the protein concentration of the lysate was determined using a BCA Protein Assay Kit (Thermo Fisher Scientific). Similar amounts of protein were then separated by sodium dodecyl sulfate‒polyacrylamide gel electrophoresis (SDS‒PAGE). The proteins were then electroblotted onto PVDF membranes and blocked with 5% TBST milk for 1-2 h. Then, the membranes were incubated overnight with primary antibodies at 4 °C. The next day, the membranes were incubated with secondary antibodies for 2 h. The protein bands were visualized by adding enhanced chemiluminescence (ECL) Western blotting substrate (Thermo Fisher Scientific).

### Immunoprecipitation (IP) and mass spectrometry (MS)

Protein lysates (0.5 mg) were incubated with 20 µL of protein G/Agarose PLUS-Agarose beads (Beckman, A63880) for 2 h. Then, the agarose was removed, and 1 μg of the indicated antibody was added and incubated for 4 h. Clean beads were added to the cell lysates, and the mixture was incubated for at least 8 h. The beads were washed six times with coimmunoprecipitation lysis buffer and subjected to Western blot analysis. To identify PLAGL2-interacting proteins, lysates from HCC cells were immunoprecipitated with a PLAGL2 antibody. The interacting proteins were analyzed by electrospray ionization tandem MS on a Thermo LTQ Orbitrap instrument. Proteins were identified with the PLAGL2 antibody. Individual ion scores are shown in the form of a Mascot-derived confidence score [calculated from the posterior error probability (PEP) as 10 log (PEP)]. The default significance threshold was a *P* value < 0.05.

### Dual-luciferase reporter assay

A luciferase reporter assay was performed using a dual-luciferase reporter assay kit (Vazyme, DL101-01). Cells in six-well plates (3 × 10^5^ cells per well) were transfected with the PLAGL2-overexpressing/zsGreen plasmid and USP10 promoter-driven luciferase construct (pGL3 basic)/*USP10* mutant-driven luciferase construct (pGL3 basic) and Renilla plasmid mixture. Luciferase activity was measured using a microplate reader.

### Chromatin immunoprecipitation (ChIP)

Chromatin immunoprecipitation (ChIP) was performed using an EZ-Magna ChIPTM A/G kit (Millipore, 17-10086) according to the manufacturer’s protocol. Briefly, HCC cells were crosslinked with 1% formaldehyde for 10 min and quenched with glycine at a final concentration of 0.2 M. Then, the cells were washed twice with ice-cold PBS and lysed with LB buffer. Triton-X 100 was added to the final lysate after sonication. The supernatant was incubated with 100 μL of protein A beads and antibodies. After cross-linking reversal and RNase treatment, DNA was extracted from the beads, eluted with ethanol, and amplified using qPCR. The primers used in the experiments are listed in Supplementary Table 2.

### Immunohistochemical (IHC) staining

Tissues were fixed in 4% paraformaldehyde, paraffin-embedded, sectioned, deparaffinized in xylene, and rehydrated in gradient ethanol. Antigen retrieval was performed using sodium citrate/TE buffer. Nonspecific binding was blocked by incubating the tissue sections with 10% goat serum in PBST. Then, the sections were incubated overnight with primary antibodies at 4 °C. After being washed, the tissues were incubated with secondary antibodies and stained with DAB. The sections were then counterstained with hematoxylin, dehydrated, and mounted. Images were obtained using a Leika DMI 3000 B microscope. Finally, protein expression was analyzed using ImageJ software.

### Ethics statement

All animal experiments were approved by the Animal Care and Use Committees of the Center for New Drug Evaluation and Research, China Pharmaceutical University (Nanjing, China) (Approval no. B20201025-2).

### Statistical analysis

Each experiment was performed in triplicate. The data were analyzed using GraphPad Prism 9.0 (GraphPad Software, Inc.) and SPSS software (version 16.0). Differences between variables were compared using two-tailed Student’s *t* tests, 1-way ANOVA, and the chi-square test, where appropriate. The following P values were used: *p* > 0.05, *p < 0.05, **p < 0.01, ***p < 0.0001, and ****p < 0.0001.

## Results

### Chronic stress promotes PLAGL2 expression and HCC progression

**Fig. 1:**
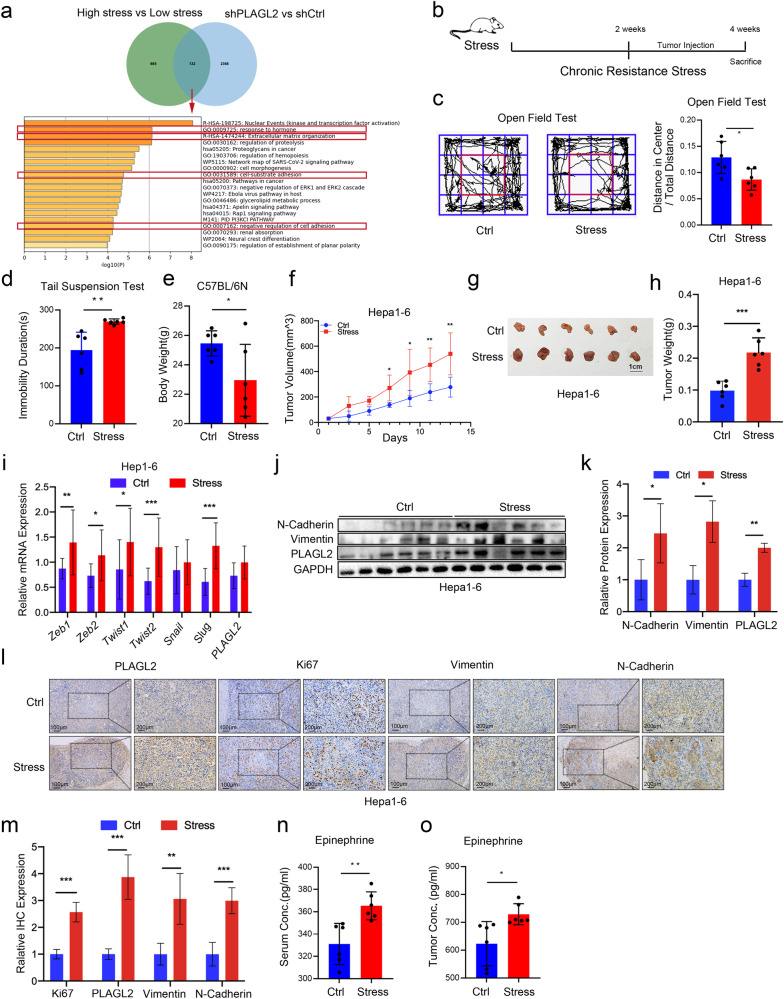
Chronic stress promotes PLAGL2 expression via epinephrine. **a** Molecular function of genes enriched in the PLAGL2 knockdown group (*p* < 0.05) and depressed (GSE9116 database, *p* < 0.05) patients, as determined by Gene Ontology enrichment analysis. **b** Schematic representation of the experimental design and timeline used to establish the chronic stress tumor mouse model. **c** Open field tests were used to assess the behaviors of chronic stress model mice (*n* = 6). **d** The tail suspension test was used to analyze the behaviors of chronic stress model mice (*n* = 6). **e** Body weights of the mice under stressed and nonstressed conditions (*n* = 6). **f** Tumor volumes of Hepa1-6 xenograft tumors in the model mice under control and stressed conditions (*n* = 6). **g**, **h** Images and weights of tumors from nonstressed and chronically stressed tumor model mice. **i** qRT‒PCR analysis of the mRNA expression of *Zeb1*, *Zeb2*, *Twist1*, *Twist2*, *Snail*, *Slug*, and *PLAGL2* in tumor tissues from model mice under control and stressed conditions. **j**, **k** Western blot and quantitative analyses of the protein levels of EMT-related factors (N-cadherin and vimentin) and PLAGL2 in tumor tissues from mice under control and stressed conditions. **l**, **m** IHC and quantitative analyses of PLAGL2, Ki67, vimentin, and N-cadherin expression in tumor tissues from control and stressed mice. **n**, **o** Concentrations (Conc.) of Epi in the serum and tumor tissue of nonstressed and chronically stressed mice were measured by ELISA. The data are presented as the mean ± SD and were analyzed by two-tailed Student’s *t* tests. *p < 0.05, **p < 0.01, ***p < 0.01. Epi epinephrine.

A previous study showed that PLAGL2 was upregulated in HCC. However, that study did not reveal the underlying molecular mechanisms^2^. In this study, we analyzed RNA sequencing data from PLAGL2 knockdown cells by Connectivity Map (CMap) analysis. A series of connective compounds related to PLAGL2 were identified. According to the connectivity score, we found that PLAGL2 was connected with various stress hormone receptor agonists/antagonists, including levodopa (a dopamine receptor agonist), isoetarine (an adrenergic receptor agonist), and metergoline (a serotonin (5-HT) and dopamine receptor antagonist) (Supplementary Fig. 1a). In addition, we analyzed differentially expressed genes in primary ovarian tumor samples from depressed and nondepressed patients in the NCBI GEO database (GSE9116). The results showed that depressed patients had higher levels of PLAGL2 (Supplementary Fig. 1b). Furthermore, the animal models revealed that mice exposed to chronic stress had higher PLAGL2 levels than nondepressed mice (Supplementary Fig. 1c). Next, we obtained 132 genes from the RNA-sequencing data of PLAGL2 knockdown cells and the GSE9116 dataset. By performing KEGG and Gene Oncology analyses, we found that the increase in PLAGL2 expression in depressed patients was associated with tumor metastasis pathways, including pathways related to extracellular matrix organization, cell-substrate adhesion, and negative regulation of cell adhesion (Fig. 1a). Taken together, these results reveal that chronic stress is associated with the upregulation of PLAGL2 and tumor metastasis.

To investigate the effects of chronic stress on PLAGL2 expression and HCC progression, we subjected C57BL6N mice to chronic restraint stress and generated a Hepa1-6 subcutaneous tumor model and a BALB/c mouse model with subcutaneous H22 tumors (Fig. 1b). The results revealed that stressed mice had more anxiety-like and depression-like behaviors (Fig. 1c, d) and lower body weights than the controls (Fig. 1e). Furthermore, the stressed mice showed faster tumor progression and higher tumor burdens than nonstressed mice (Fig. 1f–h, and Supplementary Fig. 1g). The animals were sacrificed, and the tumor tissue from stressed animals exhibited increased mRNA expression of the EMT-related genes *Twist1*, *Twist2*, *Zeb1*, *Zeb2*, *Slug*, and *PLAGL2* (Fig. 1i) and increased protein expression of PLAGL2, Ki67 (a proliferation marker), N-cadherin, and Vimentin (Fig. 1j–m, and Supplementary Fig. 1h, i). We examined the concentrations of major stress hormones, including cortisol, norepinephrine, and epinephrine. The results demonstrated that stressed mice had higher levels of epinephrine in serum and tumor tissues than control mice (Fig. 1n, o, and Supplementary Fig. 1f). However, there were no significant differences in cortisol or norepinephrine concentrations between the serum and tumor tissue of both groups (Supplementary Fig. 1d, e). Taken together, these results suggest that chronic stress upregulates the expression of PLAGL2 and promotes HCC metastasis by increasing epinephrine levels.

### Epinephrine upregulates PLAGL2 expression and promotes HCC progression

**Fig. 2:**
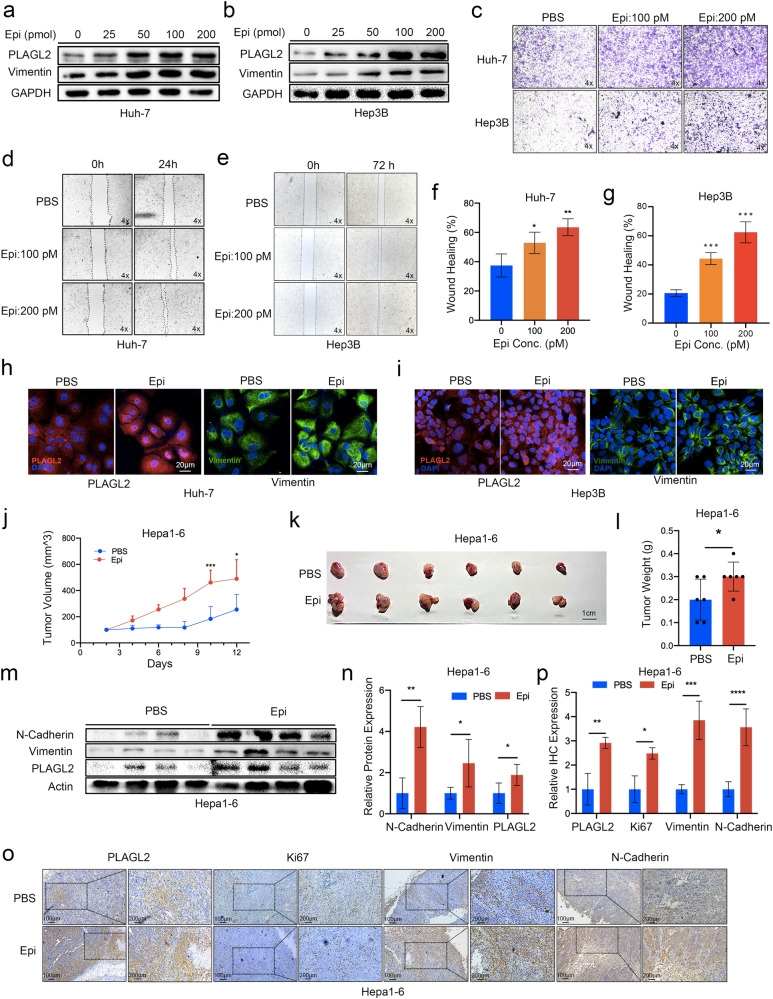
Epinephrine upregulates PLAGL2 expression and promotes HCC progression. **a**, **b** Western blot analysis of PLAGL2 and Vimentin expression in HCC cells treated with the indicated concentrations of Epi for 48 h. **c–g** Effects of Epi on the migration of HCC cells. Representative images of HCC cells in the Transwell (**c**) and wound healing assays (**d**, **e**). **f**, **g** Statistical analysis of the wound healing assay results (*n* = 3). **h**, **i** Localization and expression of PLAGL2 were detected by IF analysis of PBS- or Epi-treated HCC cells. **j** Changes in the volumes of the PBS- or Epi-treated tumors (*n* = 6). **k**, **l** Images and changes in the weights of PBS- or Epi-treated tumors (*n* = 6). **m**, **n** Western blot and quantitative analyses of the protein levels of EMT-related factors (N-cadherin and vimentin) and PLAGL2 in the tumor tissues of PBS- or Epi-treated mice. **o**, **p** IHC and quantitative analyses of PLAGL2, Ki67, vimentin, and N-cadherin expression in the tumor tissues of PBS- or Epi-treated mice. The data are presented as the mean ± SD and were analyzed by two-tailed Student’s *t* tests. *p < 0.05, **p < 0.01, ***p < 0.001, ****p < 0.0001. IF Immunofluorescence.

To further confirm the role of epinephrine, we treated HCC cells with 0, 25, 50, 100, and 200 pM epinephrine for 48 h. The results revealed that epinephrine increased the protein expression of PLAGL2 and vimentin in HCC cells in a dose-dependent manner (Fig. 2a, b) and increased the mRNA expression of EMT-related genes (Supplementary Fig. 2a, b). Furthermore, immunofluorescence staining revealed that treatment of Hep3B and Huh-7 cells with epinephrine increased the fluorescent intensities of PLAGL2 and vimentin (Fig. 2h, i). Our previous study reported that PLAGL2 promoted HCC metastasis^2^. Therefore, we explored whether epinephrine-induced PLAGL2 expression could enhance HCC metastasis. The results of transwell and wound healing assays revealed that epinephrine increased the migration of HCC cells in a dose-dependent manner (Fig. 2c–g; Supplementary Fig. 3a, b). Moreover, we investigated xenograft tumor growth in C57BL/6N mice treated with epinephrine (6 mg/kg/day s.c.). The results demonstrated that, compared with the PBS-treated group, the epinephrine-treated group had accelerated tumor growth (Fig. 2j–l). A similar result was obtained in the HCCLM3 subcutaneous tumor model in nude mice treated with epinephrine (Supplementary Fig. 4a–c). Furthermore, epinephrine was associated with increased PLAGL2 protein expression (Fig. 2m–p) and increased EMT-related mRNA and protein expression (Supplementary Fig. 2c). Taken together, these results indicate that epinephrine upregulates the expression of PLAGL2 and promotes the progression of HCC.

### Propranolol inhibits epinephrine-induced expression of PLAGL2 and HCC progression

**Fig. 3:**
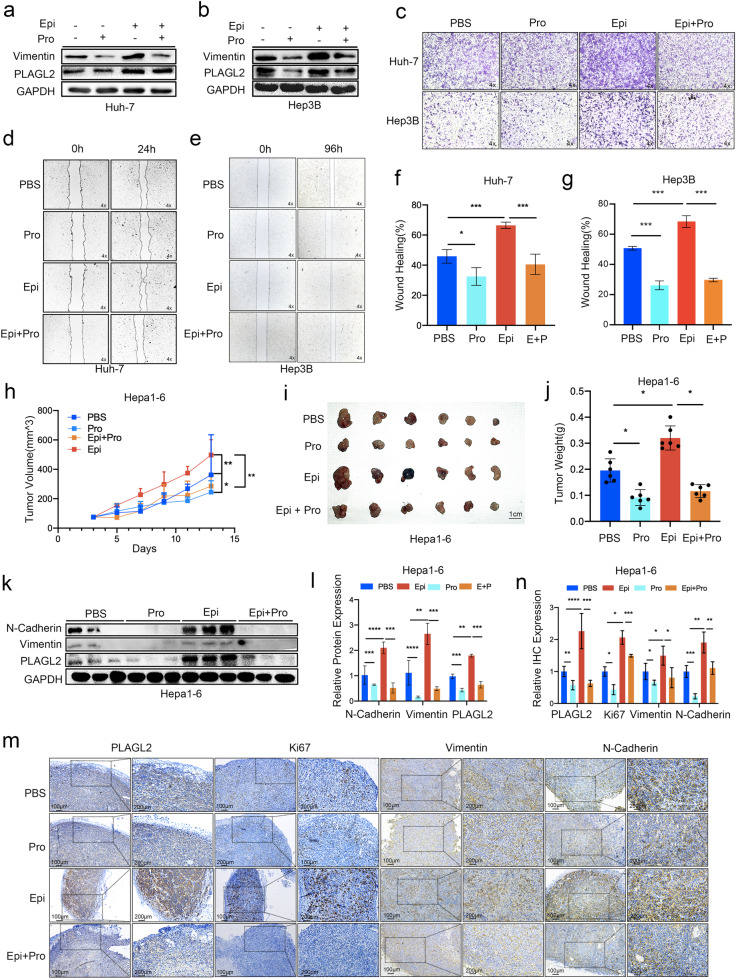
Propranolol inhibits epinephrine-mediated PLAGL2 expression and HCC progression*.* **a**, **b** HCC cells were treated with Epi (100 pM) for 48 h and then treated with propranolol (10 μM) for 24 h. The protein expression of PLAGL2 and vimentin was analyzed by Western blot analysis. **c**–**e** Effects of propranolol on the migration of HCC cells. Representative images of HCC cells in the Transwell (**c**) and wound healing assays (**d**, **e**). **f**, **g** Statistical analysis of the wound healing assay results (*n* = 3). **h** Tumor growth of Hepa1-6 cells in mice treated with Epi (2 mg/kg/d, s.c.) or propranolol (2 mg/kg/d, i.p.). **i**, **j** Images and statistical analysis of tumor weights in model mice treated with Epi and propranolol (*n* = 6). **k**, **l** Western blot and quantitative analyses of the protein levels of EMT-related factors (N-cadherin and vimentin) and PLAGL2 in the tumors of mice treated with Epi and propranolol. **m**, **n** IHC and quantitative analyses of PLAGL2, Ki67, vimentin, and N-cadherin expression in the tumors of mice treated with Epi or propranolol. The data are presented as the mean ± SD and were analyzed by two-tailed Student’s *t* tests. *p < 0.05, **p < 0.01, ***p < 0.001. Pro propranolol.

We investigated whether the increase in PLAGL2 expression was mediated by the adrenergic receptor. Treatment of HCC cells with propranolol (a nonselective β-adrenergic receptor antagonist) decreased epinephrine-induced PLAGL2 and vimentin expression (Fig. 3a, b). Furthermore, wound healing and transwell assays showed reduced cell migration in propranolol-treated HCC cells (Fig. 3c–g). In addition, treatment with propranolol was associated with reductions in tumor volume and weight with or without epinephrine treatment (Fig. 3h–j). Moreover, propranolol inhibited the epinephrine-induced expression of PLAGL2, Vimentin, and N-Cadherin (Fig. 3k–n). In conclusion, these results indicate that epinephrine upregulates the expression of PLAGL2 and promotes HCC metastasis through β-adrenergic receptors.

### PLAGL2 knockdown inhibits epinephrine-induced HCC progression

**Fig. 4:**
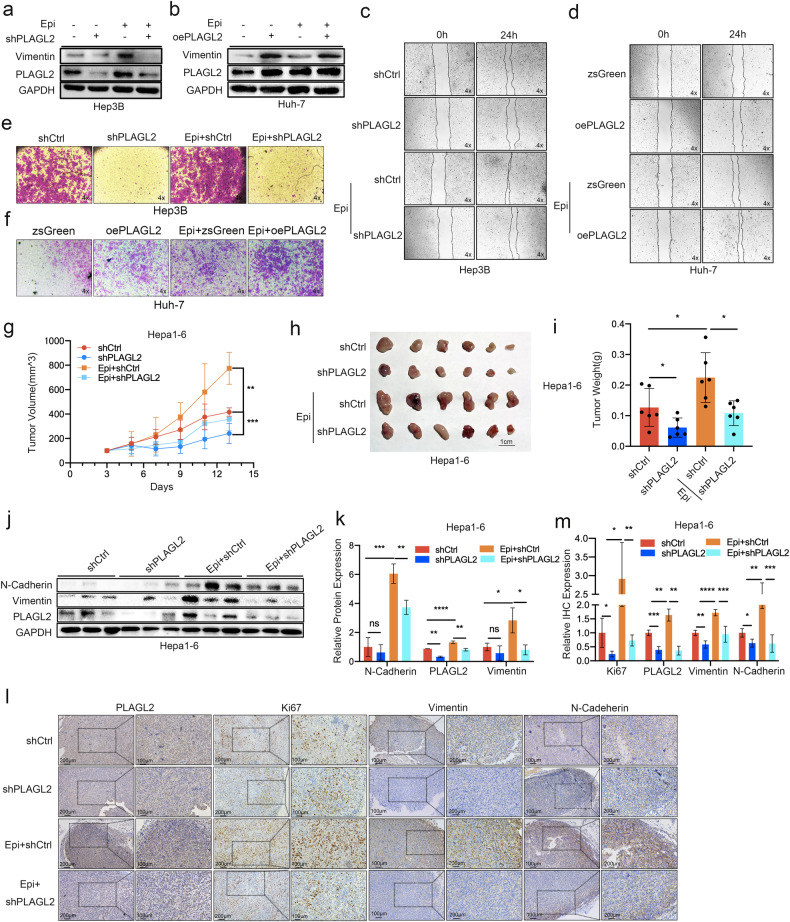
PLAGL2 knockdown inhibits Epi-induced HCC progression in vitro and in vivo. **a**, **b** PLAGL2-knockdown and PLAGL2-overexpressing HCC cells were treated with or without Epi (100 pM) for 48 h, and Western blot analysis of the protein expression of PLAGL2 and Vimentin was performed. **c**–**f** Effects of Epi on the migration of PLAGL2-knockdown or -overexpressing HCC cells. Representative images of HCC cells in the wound healing (**c**, **d**) and Transwell (**e**, **f**) assays. **g** Tumor growth of shCtrl and shPLAGL2 Hepa1-6 cells in mice treated with or without Epi (2 mg/kg/d, s.c.). **h**, **i** Images and statistical analysis of tumor weights in the shCtrl and shPLAGL2 Hepa1-6 groups treated with or without Epi. **j**, **k** Western blot and quantitative analyses of the protein levels of EMT-related factors (N-cadherin and vimentin) and PLAGL2 in shCtrl and shPLAGL2 Hepa1-6 tumors from mice treated with or without Epi. **l**, **m** IHC and quantitative analyses of PLAGL2, Ki67, vimentin, and N-cadherin expression in shCtrl and shPLAGL2 Hepa1-6 tumors in mice treated with Epi. Analyses were performed by two-tailed Student’s *t* tests. The data are presented as the mean ± SD. *p < 0.05, **p < 0.01, ***p < 0.001, ****p < 0.0001. Epi epinephrine.

We treated PLAGL2-knockdown and PLAGL2-overexpressing HCC cells with epinephrine to determine whether PLAGL2 was involved in epinephrine-induced HCC progression. As shown in Fig. 4a, b, PLAGL2-knockdown cells exhibited decreased expression of Vimentin, while PLAGL2-overexpressing cells exhibited significantly increased expression of Vimentin. Furthermore, PLAGL2-knockdown cells exhibited decreased migration, while PLAGL2-overexpressing cells exhibited increased migration (Fig. 4c–f, Supplementary Figs. 5a, b and 6a, b). Moreover, PLAGL2 knockdown in tumors was associated with reduced sensitivity to epinephrine. The shPLAGL2 group exhibited decreases in tumor volume and tumor mass following epinephrine treatment (Fig. 4g–i). In addition, PLAGL2 inhibition was associated with decreased expression of PLAGL2, vimentin, and N-cadherin following treatment with epinephrine (Fig. 4j–m).

**Fig. 5:**
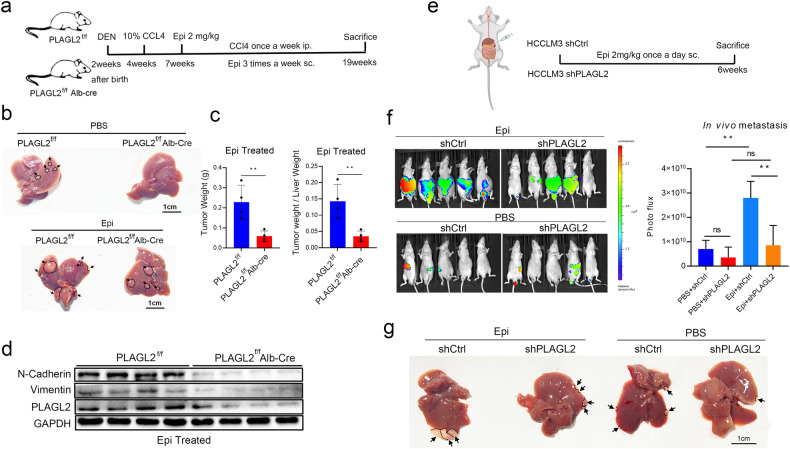
Hepatocyte-specific PLAGL2 knockout suppresses epinephrine-induced HCC tumorigenesis and metastasis. **a** Schematic representation of DEN/CCl4-induced HCC models treated with PBS or Epi (2 mg/kg, 3 times/week). **b** Images of the livers of DEN/CCl4-induced HCC model mice. Arrowheads indicate tumor nodules in the liver. **c** Total and relative weights of tumor tissues from PLAGL2 ^f/f^ and PLAGL2 ^f/f^ Alb-cre mice treated with PBS or Epi. Analyses were performed by two-tailed Student’s *t*-test (*n* = 4). **d** Western blot analysis of the protein levels of EMT-related factors (N-cadherin and vimentin) and PLAGL2 in the tumors of PLAGL2 ^f/f^ and PLAGL2 ^f/f^ Alb-cre mice treated with PBS or Epi. **e** Schematic representation of liver metastasis mouse models induced by intrasplenic injection of shPLAGL2 and shCtrl HCCLM3 cells. **f** Representative in vivo images of the spleens of mice injected with the indicated HCCLM3 cells. **g** Representative images of the livers of mice treated with PBS or Epi (2 mg/kg/d). The data are presented as the mean ± SD. *p < 0.05.

We used hepatocyte-specific deletion of PLAGL2 in mice (PLAGL2^f/f^ Alb-cre) to validate whether knocking down PLAGL2 could inhibit epinephrine-induced tumorigenesis and tumor progression in vivo. An N-nitrosodiethylamine (DEN)/carbon tetrachloride (CCl4)-induced HCC mouse model was established (Fig. 5a). The results revealed that epinephrine enhanced tumor progression in the PLAGL2^f/f^ and PLAGL2^f/f^ Alb-cre groups. Compared with the PLAGL2^f/f^ epinephrine-treated group, the PLAGL2^f/f^ Alb-cre epinephrine-treated group exhibited reductions in tumor nodules and tumor weight (Fig. 5b, c). In addition, the PLAGL2^f/f^ Alb-cre epinephrine-treated group showed decreased expression of Vimentin and N-Cadherin compared with the PLAGL2^f/f^ epinephrine-treated group (Fig. 5d). Furthermore, the in vivo metastasis model showed that PLAGL2 knockdown was associated with reduced metastasis of HCC cells from the spleen to the liver with or without epinephrine treatment (Fig. 5e–g). Taken together, these results indicate that PLAGL2 knockdown inhibits epinephrine-induced HCC development and metastasis. Furthermore, these results reveal that epinephrine promotes HCC progression via PLAGL2.

### Epinephrine stabilizes PLAGL2 via USP10

**Fig. 6:**
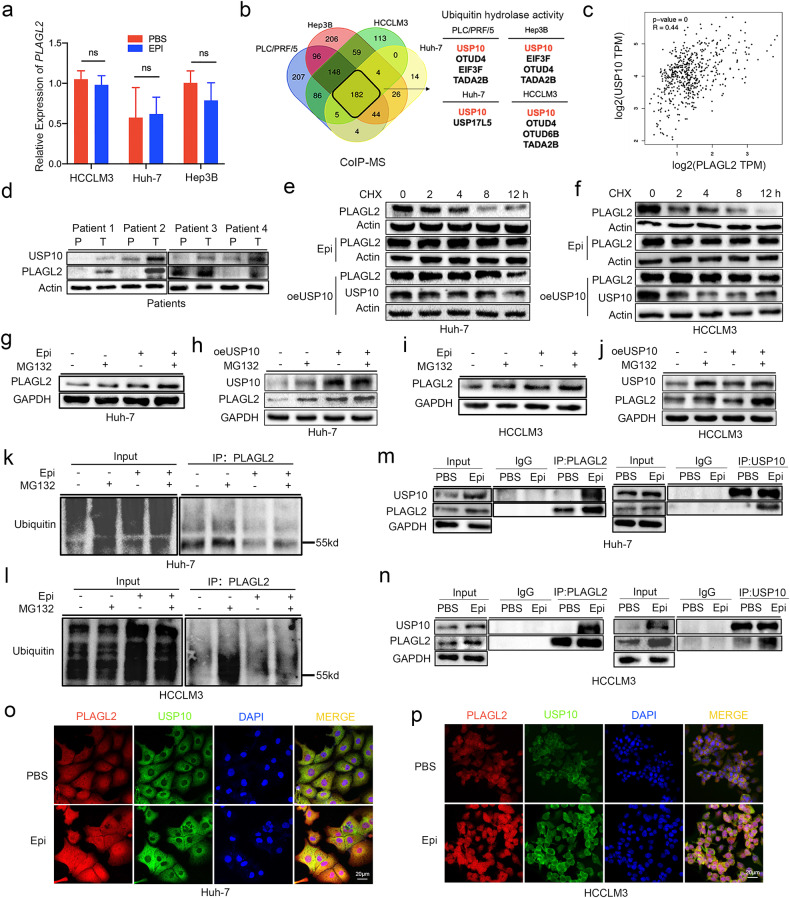
Epinephrine-induced USP10 deubiquitinates and stabilizes PLAGL2. **a** The mRNA expression of *PLAGL2* in HCC cells was measured by RT‒qPCR (*n* = 3). HCC cells were treated with epinephrine (100 pM) for 48 h. **b** Venn plot of peptides with ubiquitin hydrolase activity that interact with PLAGL2 in the four HCC cell lines according to co-IP and mass spectrometry. **c** Correlations between PLAGL2 and USP10 expression in the LIHC cohort of TCGA patients were determined by Pearson’s correlation analysis. **d** Expression of PLAGL2 and USP10 in HCC patients was verified by Western blotting (P, precancerous tissues; T, tumor tissues). **e**, **f** Western blot analysis of the protein levels of PLAGL2 in HCC cells treated with Epi (100 pM) for 48 h or transfected with the USP10 overexpression (oeUSP10) plasmid followed by treatment with cycloheximide (CHX) (100 μg/ml) for the indicated times. **g**–**j** Western blot analysis of the protein levels of PLAGL2 in HCC cells treated with Epi (100 pM) or transfected with the oeUSP10 plasmid followed by treatment with MG132 (10 μM). **k**, **l** The levels of PLAGL2 ubiquitination in anti-PLAGL2 immunoprecipitates and whole-cell lysates (input) derived from Huh-7 and HCCLM3 cells treated with Epi (100 pM) for 48 h and MG132 (10 μM) for 12 h. **m**, **n** Co-IP assays of HCC cells treated with or without Epi (100 pM), USP10, or PLAGL2 and immunoprecipitated with anti-PLAGL2 and anti-USP10 antibodies. **o**, **p** The localization and expression of PLAGL2 and USP10 in PBS- or Epi-treated HCC cells were examined by IF assays. Epi epinephrine.

To examine the effects of epinephrine on PLAGL2 expression, we analyzed *PLAGL2* mRNA expression following epinephrine treatment. Epinephrine induced no changes in the mRNA expression of *PLAGL2* (Fig. 6a). Therefore, we hypothesized that epinephrine affected PLAGL2 expression via posttranslational modification. We performed coimmunoprecipitation mass spectrometry (co-IP MS) to analyze PLAGL2-interacting peptides in the extracts of four types of HCC cells. The results revealed USP10 as the most likely candidate associated with PLAGL2 upregulation (Fig. 6b). In addition, an examination of HCC patients revealed that PLAGL2 expression was positively correlated with USP10 expression (Fig. 6c, d). The chronic stress mice model was examined and showed significantly increased expression of USP10 and PLAGL2 in xenograft tumors. Furthermore, immunofluorescence staining of Huh-7 and HCCLM3 cells showed that epinephrine enhanced the fluorescence intensity of USP10 (Supplementary Fig. 7a, b).

To investigate whether epinephrine and USP10 inhibited PLAGL2 protein degradation, we treated HCC cells with the protein synthesis inhibitor cycloheximide (CHX) (Fig. 6e, f, and Supplementary Fig. 7c, d). The PBS-treated group showed decreased PLAGL2 protein expression. However, PLAGL2 degradation was significantly attenuated in the presence of epinephrine and USP10. These results indicated that epinephrine enhanced the stability of PLAGL2. Next, we treated the cells with the proteasome inhibitor MG132. MG132 increased the protein expression of PLAGL2 in the presence of epinephrine (Fig. 6g, i) and USP10-overexpression plasmid (Fig. 6h, j), suggesting that epinephrine stabilized PLAGL2 via USP10. Next, we treated HCC cells with epinephrine and MG132. Ubiquitination assays revealed that ubiquitin enrichment on PLAGL2 (55 kDa) was significantly reduced following epinephrine treatment (Fig. 6k, l). Co-IP and immunofluorescence staining showed that epinephrine promoted the binding of PLAGL2 to USP10 (Fig. 6m–p). These results suggest that epinephrine stabilizes PLAGL2 by eliminating its ubiquitination via USP10.

### PLAGL2 activates USP10 transcription

**Fig. 7:**
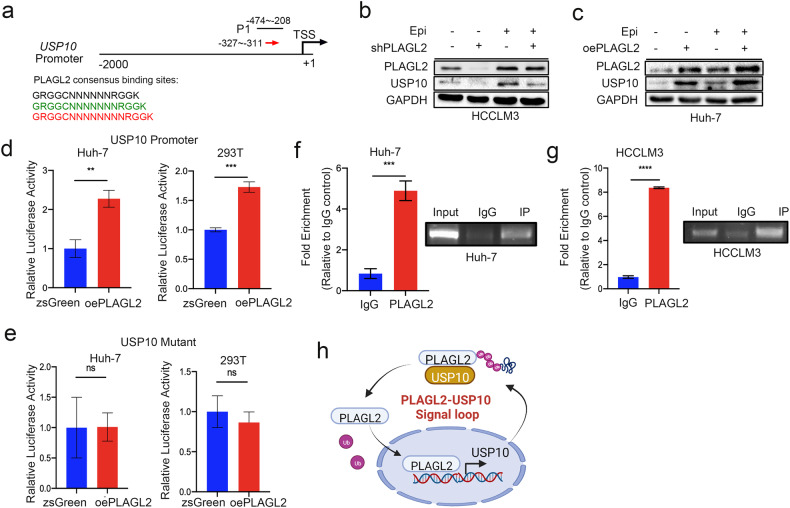
PLAGL2 functions as a transcription factor of USP10. **a** PLAGL2 consensus binding sites were identified in the USP10 promoter (−2000 to +1 from the translation start site). These sites were identified as GRGGC (NNNNNN)RGGK (black arrow), GRGGC (NNNNNNN)RGGK (green arrow), and GRGGC(NNNNNNNN)RGGK (red arrow). **b**, **c** Western blot analysis of the protein levels of PLAGL2 and USP10 in PLAGL2-knockdown and PLAGL2-overexpressing HCC cells treated with or without Epi (100 pM). **d** The regulatory effects of PLAGL2 on the USP10 promoter in Huh-7 and 293T cells were examined by dual-luciferase reporter assays. **e** The regulatory effects of PLAGL2 on the USP10 mutant promoter in Huh-7 and 293T cells were examined by dual-luciferase reporter assays. **f**, **g** Enrichment of PLAGL2 in the USP10 promoter region in Huh-7 and HCCLM3 cells was determined by ChIP assays. **h** Model of the epinephrine-induced USP10-PLAGL2 signaling loop. The data are presented as the mean ± SD. *p < 0.05, **p < 0.01, ***p < 0.001, ****p < 0.0001. Epi epinephrine.

PLAGL2 has been shown to activate transcription by binding to the GRGGC(N)_6-8_RGGK consensus site in regulatory regions of target genes. In this study, we identified a PLAGL2 consensus binding site (−327 to −311 bp) in the USP10 promoter region (−2000 to +1 from the translation start site), as shown in Fig. 7a. Overexpression of PLAGL2 increased the protein expression of USP10 with or without epinephrine treatment. However, PLAGL2 knockdown decreased the protein expression of USP10 in cells treated with or without epinephrine (Fig. 7b, c). To explore the binding sites of PLAGL2 associated with the transactivation of *USP10*, we designed a USP10 promoter and USP10 promoter mutants and subcloned them into a luciferase vector. To further investigate whether PLAGL2 directly activated USP10 transcription, we transfected HEK293T and Huh-7 cells with PLAGL2-overexpressing plasmids and a luciferase reporter carrying the USP10 promoter sequence. HEK293T and Huh-7 cells exhibited significant increases in luciferase reporter activity (Fig. 7d). However, mutations in the consensus PLAGL2 binding sites abolished PLAGL2-mediated USP10 transcription (Fig. 7e). Moreover, a quantitative chromatin immunoprecipitation (qChIP) assay was performed to examine the enrichment of the PLAGL2 antibody in the USP10 promoter in Huh-7 and HCCLM3 cells. The qChIP assay demonstrated that PLAGL2 and USP10 could bind to the −474 to −208 promoter site (Fig. 7f, g). These results reveal that PLAGL2 directly interacts with the USP10 promoter region and functions as a transcriptional regulator of USP10, while USP10 deubiquitinates PLAGL2 to stabilize PLAGL2’s protein expression, both of which form a signaling loop to enhance HCC progress (Fig. 7h).

### The epinephrine-induced ADRB2-c-Myc axis activates the USP10-PLAGL2 signaling loop

**Fig. 8:**
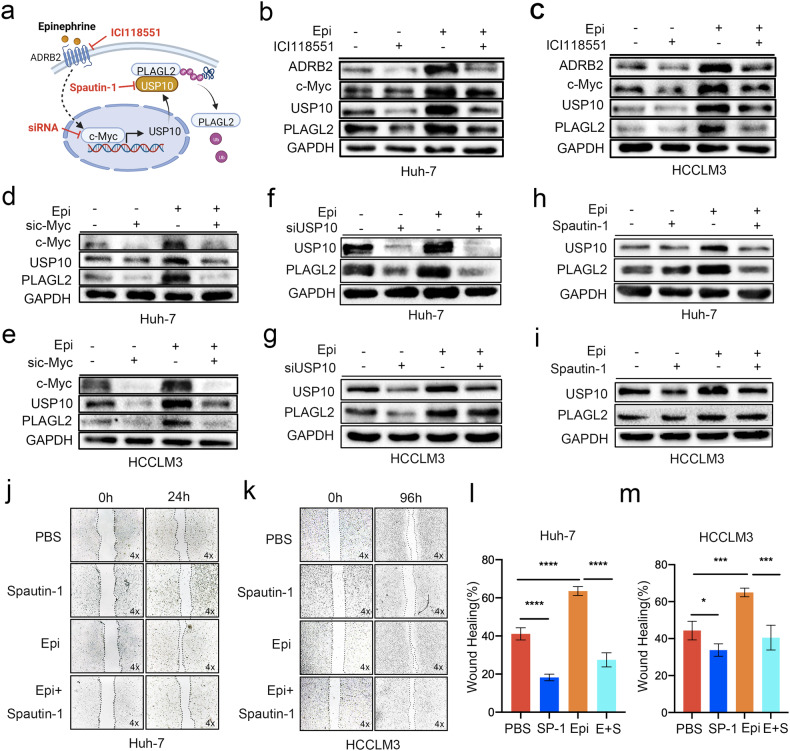
Epinephrine induces the ADRB2-c-Myc axis to activate USP10 transcription and promote HCC progression. **a** Model of Epi-induced USP10-PLAGL2 signaling loop activation via ADRB2-c-Myc. **b**, **c** HCC cells were treated with ICI118, 551 (10 μM) or Epi (100 pM) for 48 h, after which Western blot analysis was performed to measure the protein levels of ADRB2, c-Myc, USP10, and PLAGL2. **d**, **e** HCC cells were transfected with c-Myc siRNA (si465) for 24 h and treated with Epi (100 pM) for 48 h, after which Western blot analysis was performed to determine the protein levels of c-Myc, USP10 and PLAGL2. **f**, **g** HCC cells were transfected with USP10 siRNA (si1756) for 24 h and treated with Epi (100 pM) for 48 h, after which Western blot analysis was performed to determine the protein levels of USP10 and PLAGL2. **h**, **i** HCC cells were treated with the USP10-specific antagonist Spautin-1 (10 μM) or Epi (100 pM) for 48 h, after which Western blot analysis was performed to measure the protein levels of USP10 and PLAGL2. **j**–**m** Representative images of HCC cells treated with Spautin-1 (10 μM) or Epi (100 pM) for 24 h or 96 h and examined by a wound healing assay. The data are presented as the mean ± SD. *p < 0.05, **p < 0.01, ***p < 0.001, ****p < 0.0001. Epi epinephrine.

**Fig. 9:**
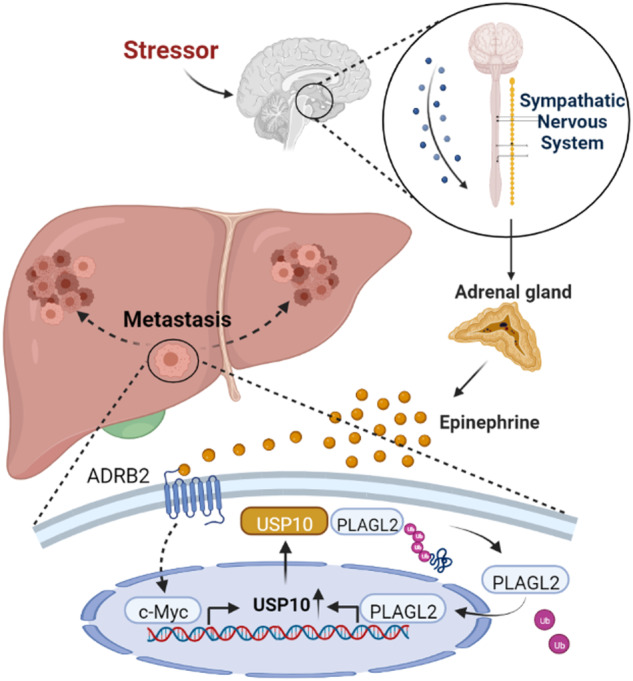
Schematic depiction of the Epinephrine-ADRB2-c-Myc-USP10-PLAGL2 interaction in HCC cells. Schematic representation showing how stress-induced epinephrine increases the expression of PLAGL2 through the ADRB2-c-Myc-USP10 signaling axis to promote the metastasis and progression of HCC.

The upstream targets responsible for the increase in the expression of the PLAGL2-USP10 signaling loop were investigated. We analyzed the USP10 promoter sequence with the JASPAR database, and MYC was identified as a candidate for USP10 transactivation. Aram et al. reported that c-Myc was a transcriptional activator of USP10 that bound to the second E-box upstream of the USP10 transcription start site^16^. Previous studies have shown that epinephrine activates ADRB2 to alter USP28-mediated deubiquitylation and stabilization of c-Myc^17,18^ (Fig. 8a). To validate this signaling axis, we treated HCC cells with ICI 118,551, which is an ADRB2 antagonist. Treatment of HCC cells with ICI 118,551 reduced the expression of c-Myc, USP10, and PLAGL2 (Fig. 8b, c). Furthermore, c-Myc knockdown by a small interfering RNA (siRNA) inhibited the epinephrine-induced expression of USP10 and PLAGL2 (Fig. 8d, e, and Supplementary Fig. 8a). Furthermore, inhibiting USP10 with Spautin-1 or siRNA significantly decreased the expression of PLAGL2 (Fig. 8f–i and Supplementary Fig. 8b). Moreover, inhibiting USP10 expression significantly reduced the migration of HCC cells induced by epinephrine (Fig. 8j–m). Taken together, these findings suggest that chronic stress-induced epinephrine activates the ADRB2-c-Myc axis, thereby upregulating USP10 to stabilize PLAGL2, while PLAGL2 acted as a transcriptional regulator of USP10, forming a signaling loop to promote HCC progression (Fig. 9).

## Discussion

Chronic stress activates the sympathetic nervous system (SNS) and the hypothalamic‒pituitary‒adrenal (HPA) axis. In addition, chronic stress is associated with cell proliferation, the disruption of phenotypic plasticity, cell death resistance, increased genomic instability and mutations, the activation of tumor-promoting inflammation, immune escape, and increased tumor invasion and metastasis^19,20^. Animal experiments demonstrated that chronic stress promoted tumorigenesis^21,22^ and tumor metastasis^23,24^. Furthermore, multiple clinical trials have shown that patients with elevated serum/urinary levels of catecholamines^25,26^ and increased expression of beta-adrenergic receptors have a poor prognosis^27,28^.

This study revealed that chronic stress promoted HCC progression via an epinephrine-induced USP10-PLAGL2 signaling loop. Furthermore, PLAGL2 was shown to promote tumor progression and metastasis, which was consistent with the findings of a previous study^2^. CMap analysis, GO, KEGG analysis of GSE9116, and the chronic stress model demonstrated that chronic stress increased the expression of PLAGL2. In vitro and in vivo assays showed that stress-induced epinephrine was associated with increased PLAGL2 expression. However, treatment of HCC cells with propranolol (a nonselective beta-adrenergic antagonist) decreased the expression of PLAGL2. Furthermore, PLAGL2 knockdown could inhibit epinephrine-induced HCC progression. On the other hand, co-IP and immunofluorescence analyses showed that the ubiquitin hydrolysis peptide ubiquitin-specific peptide (USP10) was the protein responsible for the epinephrine-induced stabilization of PLAGL2. The results revealed that epinephrine upregulated the expression of USP10 via the ADRB2-c-Myc axis. By treating HCC cells with the protein synthesis inhibitor cycloheximide and the proteasome inhibitor MG132, we showed that epinephrine-induced USP10 stabilized PLAGL2 via deubiquitylation. In addition, the present study revealed that PLAGL2 was a transcriptional regulator of USP10. Stress-induced epinephrine could activate the PLAGL2-USP10 signaling loop, thus promoting HCC progression. Taken together, the results of the present study show that epinephrine increases the expression of PLAGL2. Therefore, PLAGL2 can be used as a potential therapeutic target in HCC.

Multiple studies have shown that chronic stress promotes tumor metastasis^29,30^. A previous study demonstrated that depression promoted tumor metastasis in prostate cancer patients^31^. GSEA of the GSE9116 cohort of ovarian cancer patients revealed the enrichment of genes related to depression that were involved in tumor metastasis-related signaling pathways, such as extracellular matrix adhesion and cell migration^32^. Mechanistically, stress-induced hormones promote remodeling of the tumor microenvironment, tumor-related angiogenesis, and lymphangiogenesis^33^. Norepinephrine was shown to promote breast cancer metastasis^34^. Researchers demonstrated increased colonization and tumor activation of glucocorticoid receptors by stress hormones at distant metastatic sites in breast cancer patients^35^. This study focused on stress-induced epinephrine, which upregulated the expression of PLAGL2, thus promoting HCC metastasis. Epinephrine promotes the synthesis of heparan sulfate by upregulating the expression of 6-O-sulfotransferase-1 (6-OST-1) through the Src-ERK1/2 signaling pathway. Furthermore, epinephrine increases the activity of murine fibroblasts, thus promoting tumor metastasis^36^. However, recent evidence shows that exercise-induced epinephrine could suppress tumor growth^37^. A cohort study of 1.44 million people from Europe and the United States of America revealed that moderate and high-intensity exercise was associated with a decreased risk of various cancers, including breast, colon, rectal, esophageal, lung, liver, renal, bladder, and head and neck cancers^38,39^. Additionally, β2-AR/CCL2 activation was associated with decreased PD-L1 resistance and reduced tumor burdens in eustress mouse models subjected to exercise with running wheels. In addition, the results demonstrated that low concentrations of epinephrine and norepinephrine inhibited tumor growth. However, high concentrations of epinephrine and norepinephrine were shown to promote tumor growth. A U-shaped relationship was revealed between epinephrine/norepinephrine concentrations and CCL2 expression^40^. The differential effects of different concentrations of epinephrine/norepinephrine could be due to the activation of various adrenergic receptor subtypes, such as ADRB1, ADRB2, and ADRB3, or the complex regulatory mechanisms of ADRBs.

Ubiquitination is an essential posttranslational modification that regulates the stability and function of proteins by attaching ubiquitin (Ub) to the target protein. However, deubiquitinating enzymes (DUBs) cleave Ub from target proteins, thus reversing ubiquitination. A previous study revealed that chronic stress-induced epinephrine promoted the deubiquitylation of MYC by USP28 via lactate dehydrogenase A (LDHA)-dependent metabolic reprogramming in breast cancer. In addition, chronic stress-induced epinephrine activated the SLUG promoter and promoted stem cell-like features in breast cancer^17^. The USP10 protein contains 798 amino acids (aa) and is found in the nucleus and cytoplasm of almost all cells^41^. USP10 is a member of the mammalian ubiquitin-specific peptidase family. However, the role of USP10 in cancer regulation is controversial. The present study demonstrated that USP10 stabilized the transcription factor PLAGL2, thus enhancing HCC metastasis. Furthermore, this study demonstrated that PLAGL2 activated the transcription of USP10, forming a signaling loop. A recent study showed that USP10 promoted the progression of HCC by stabilizing YAP/TAZ^42^. Furthermore, Yang et al. reported that USP10 promoted HCC cell migration and invasion by stabilizing the Smad4 protein^43^. However, the role of USP10 in tumor progression remains controversial. Lin Z et al. showed that USP10 inhibited tumor formation by antagonizing the transcriptional activation of c-Myc through SIRT6^18^. Furthermore, USP10 inhibited lung cancer cell growth and invasion by upregulating PTEN^44^. Therefore, the role of USP10 in tumor development needs to be further investigated.

This study revealed that chronic stress-induced epinephrine increased the expression of PLAGL2, thus promoting the metastasis and progression of HCC. Therefore, PLAGL2 is a promising therapeutic target in HCC.

### Supplementary information

Supplementary information
