# Supplementary material for: Stress-induced epinephrine promotes hepatocellular carcinoma progression via the USP10-PLAGL2 signaling loop
Source: Exp Mol Med. 2024 May 1;56(5):1150–63. doi: 10.1038/s12276-024-01223-0 (PMC11148159; doi:10.1038/s12276-024-01223-0)
Supplement: Supplementary file 1 — Supplementary information [file 12276_2024_1223_MOESM1_ESM.pdf]

Supplementary Information

Stress-induced Epinephrine Promotes Hepatocellular Carcinoma Progression via the USP10-PLAGL2 signaling loop

Chen Wang[1], Jiaping Ni[1], Dongqing Zhai[1], Yanchao Xu[3], Zijie Wu[1], Yuyuan Chen[1], Ning Liu[3], Juan Du[3], Yumeng Shen[1], Yong Yang[1*], Linjun You[1*], Weiwei Hu[1,2*].

Supplementary Information

Supplementary Fig. 1-8

Supplementary Table 1-5

Supplementary Fig. 1-8

Supplementary Fig. 1

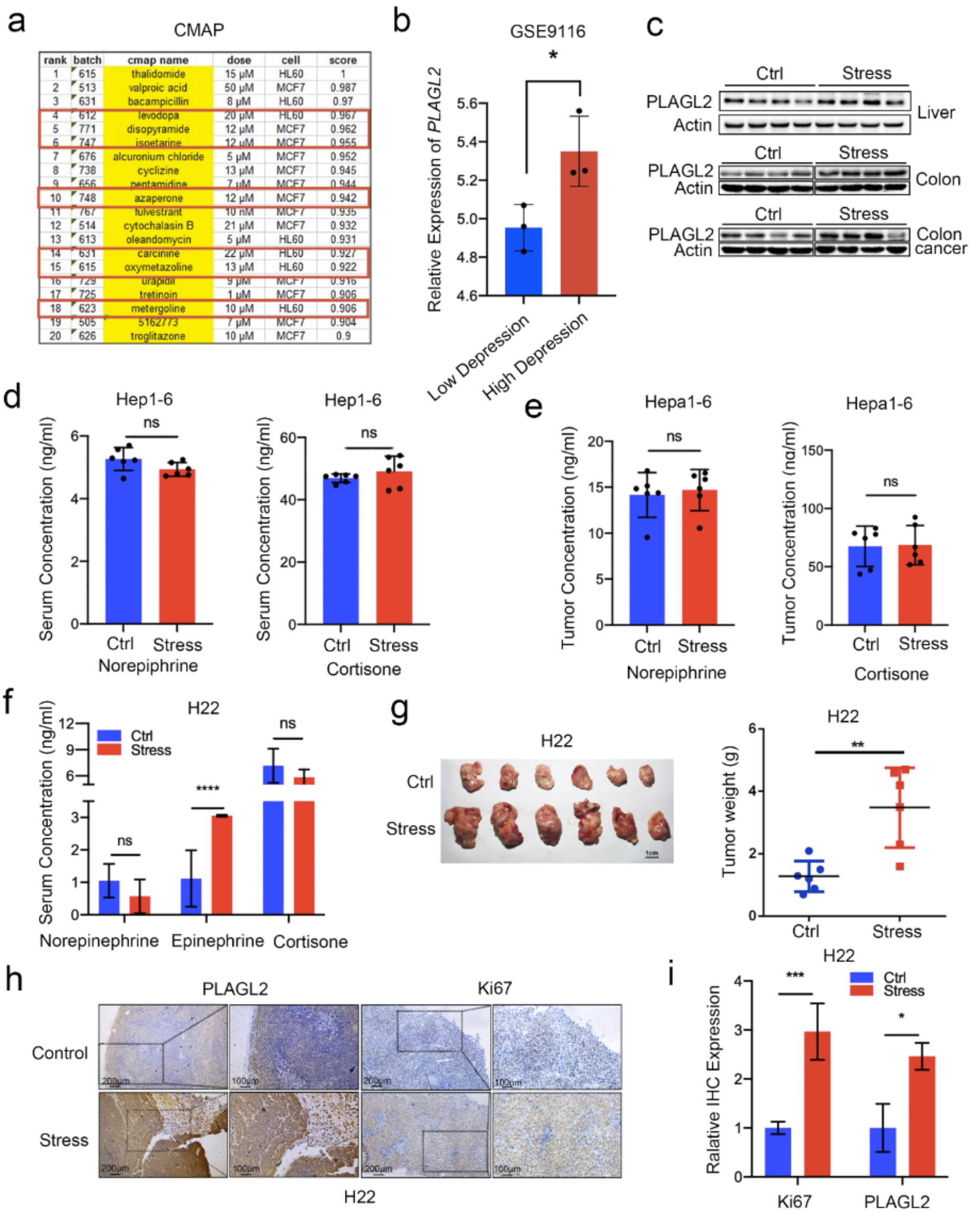

Supplementary Fig. 1. Chronic stress upregulates PLAGL2 expression. a Differentially expressed genes from PLAGL2 knockdown were analyzed by the CMAP database. **b** PLAGL2 expressions in low and high depressed patients from the GSE9116 database (n = 3). **c** Western blot analysis for protein levels of PLAGL2 in tissues (liver in the first line, colon in the second and colon tumor in the third line)

from chronically stressed mice models (n = 4). **d-f** Concentrations of Norepinephrine, Epinephrine and Cortisone in serum and tumor tissues from non-stressed and chronically stressed mice models were measured by ELISA. (n = 6). **g** Tumor images and weights from non-stressed and chronically stressed group. **h**, **i** IHC and quantitative analyses of PLAGL2 and Ki67 expressions in tumor tissues from mice in control and stressed conditions. Data are presented as mean ± SD and were analyzed by two-tailed Student's *t*-tests. *$p < 0.05$, **$p < 0.01$, ***$p < 0.001$, ****$p < 0.0001$. CAMP, Complement Map Database. Epi, epinephrine.

Supplementary Fig. 2

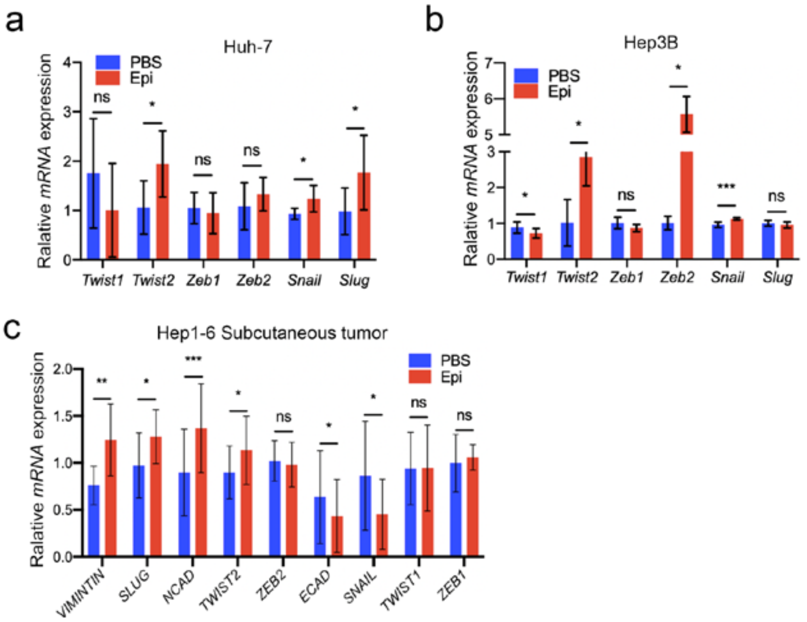

Supplementary Fig. 2 Epinephrine upregulates EMT-related genes expression. a, **b** qRT-PCR analysis for Twist1, Twist2, Zeb1, Zeb2, Snail, and Slug mRNA expression in HCC cells treated with Epi (100 pmol) for 24 h. **c** qRT-PCR analysis for Vimentin, Slug, N-cadherin, Twist2, Zeb2, E-cadherin, Snail, Twist1 and Zeb1 mRNA expressions in tumor tissues from mice treated with PBS or Epi. Data are presented as mean ± SD. *p < 0.05, **p < 0.01, ***p < 0.001. Epi, epinephrine.

Supplementary Fig. 3

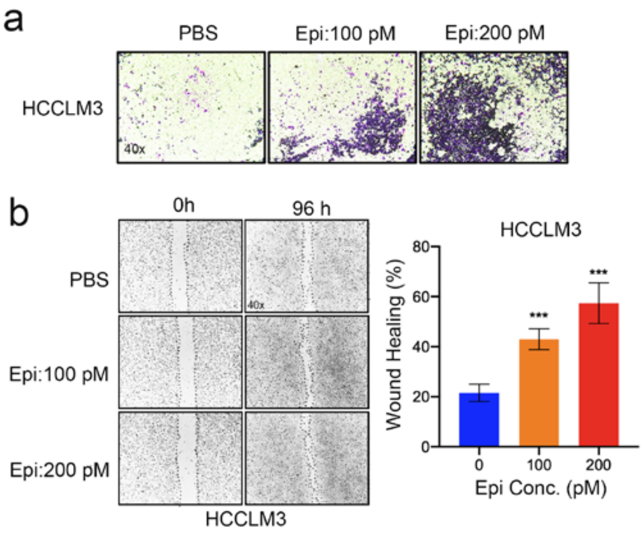

Supplementary Fig. 3 Epinephrine promotes HCCLM3 cell migration *in vitro*. a Effects of Epi on migration abilities of HCC cells, representative images of HCC cells in the Transwell. **b** Analysis of wound healing abilities of HCCLM3 cell treated with/without Epi (100 pM) (n = 3). Data are presented as mean ± SD. *$p < 0.05$, **$p < 0.01$, ***$p < 0.001$, ****$p < 0.0001$. Epi, epinephrine.

Supplementary Fig. 4

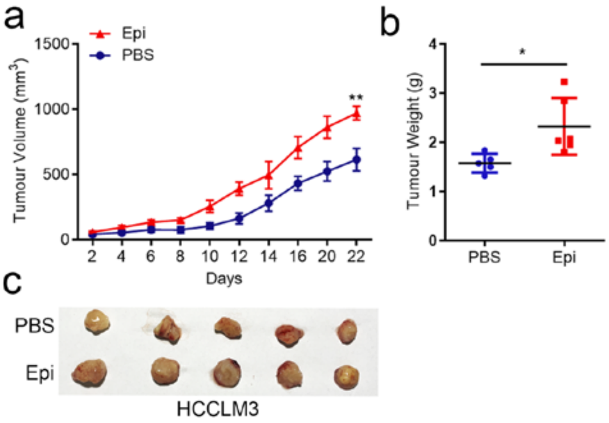

Supplementary Fig. 4 Epinephrine promotes HCCLM3 cell proliferation *in vivo*. **a** Changes in volumes of PBS- or Epi-treated (6mg/kg) tumors (n = 5). **b c** Image and changes in weights of PBS- or Epi-treated tumors (n = 5). Data are presented as mean ± SD. *$p < 0.05$, **$p < 0.01$. Epi, epinephrine.

Supplementary Fig. 5

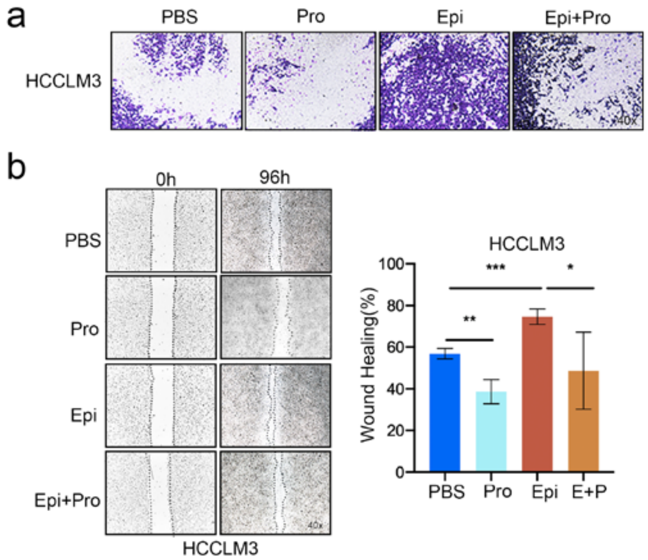

Supplementary Fig. 5 Propranolol inhibits Epinephrine-dependent HCCLM3 cell migration. a, b Effects of propranolol on migration abilities of HCCLM3 cell, representative images of HCC cells in Transwell (a) and wound healing assays (b) (n = 3). Data are presented as mean ± SD. *p < 0.05, **p < 0.01, ***p< 0.001. Epi, epinephrine.

Supplementary Fig. 6

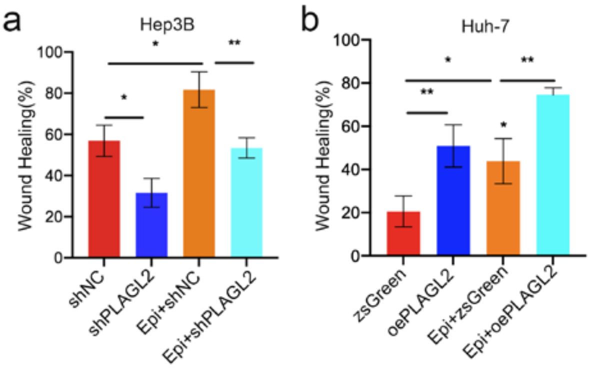

Supplementary Fig. 6 PLAGL2 is a key regulator in Epi-induced HCC metastasis. **a, b** Analysis of wound healing abilities of PLAGL2 knockdown or overexpression HCC cells treated with/without Epi (100 pM) (n = 3). Data are presented as mean ± SD. *$p < 0.05$, **$p < 0.01$, ***$p < 0.001$. Epi, epinephrine.

Supplementary Fig. 7

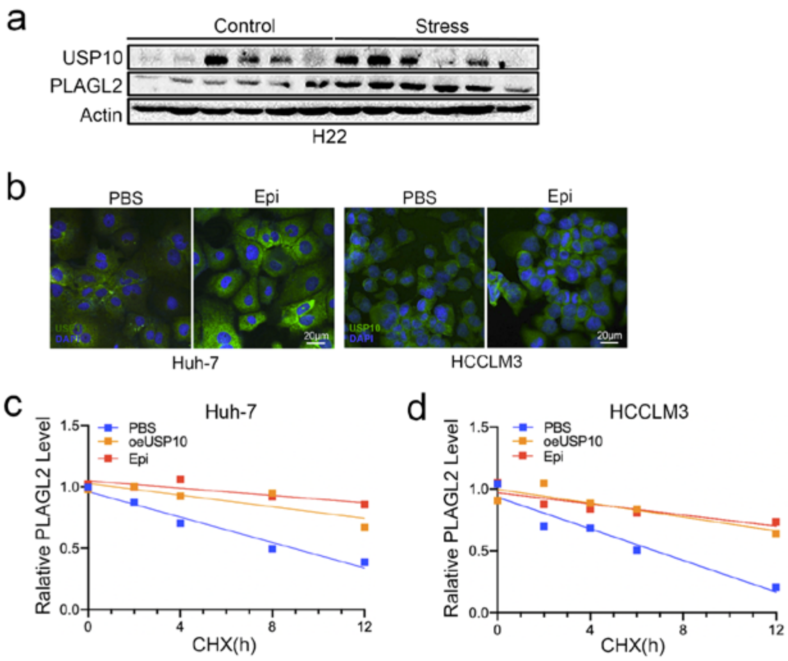

Supplementary Fig. 7 Epinephrine-activated USP10 interacts with PLAGL2 physically. a Western blot analysis of the relevance between USP10 and PLAGL2 in a Stress-induced subcutaneous tumor model (H22). **b** IF assays detected the expression of USP10 in PBS or Epi-treated HCC cells. **c, d** The intensity of PLAGL2 expressions was quantified and plotted against time

Supplementary Fig. 8

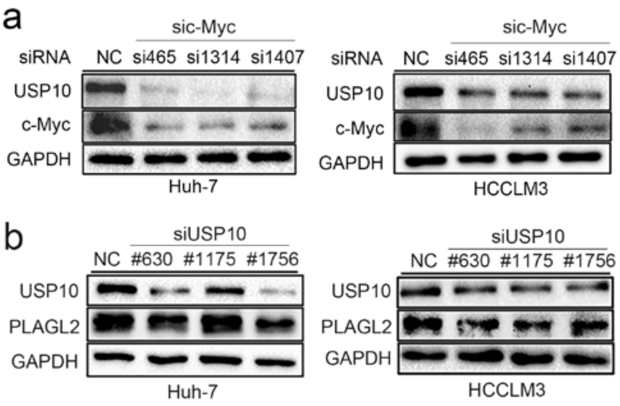

Supplementary Fig. 8 Validation of USP10 and c-Myc siRNA efficient. a, b HCC cells were transfected with 3 different siRNAs targeting c-Myc (**a**) and USP10 (**b**).

Supplementary Table 1-5

Supplementary Table 1. The primers have been used for Real-time PCR

Name	Sequence（5’-3’）
Human-PLAGL2-F	CCAGAGCAGAGACCATATAG
Human-PLAGL2-R	AACATCTTATCACAGTACATACAC
Human-E-Cadherin-F	CGAGAGCTACACGTTCACGG
Human-E-Cadherin-R	GGGTGTCGAGGGAAAAATAGG
Human -N-Cadherin-F	TCAGGCGTCTGTAGAGGCTT
Human -N-Cadherin-R	CCAGTCTTGCATAATGCGATTTC
Human-Vimentin-F	GAAAAACCTTCCCGGTGCAAT
Human-Vimentin-R	TGTCTTTGCTCGAATGTGCG
Human-Snail-F	TAGCGAGTGGTTCTTCTGCG
Human-Snail-R	AGGGCTGCTGGAAGGTAAAC
Human-Slug-F	AAGCATTTCAACGCCTCCAAA
Human-Slug-R	GGATCTCTGGTTGTGGTATGACA
Human-Zeb1-F	CTACAACAACAAGACACTGCTGT
Human-Zeb1-R	TGTTCTTTCAGAGAGGTAAAGCG
Human-Zeb2-F	CAAGAGGCGCAAACAAGCC
Human-Zeb2-R	GGTTGGCAATACCGTCATCC
Human-Twist1-F	AGAGGTCGTGCCAATCAGC
Human-Twist1-R	ACTATGGTTTTGCAGGCCAGT
Human-Twist2-F	GAGCGACGAGATGGACAATAAGA
Human-Twist2-R	ATGCGCCACACGGAGAA
Human-actin-F	GCGTGACATTAAGGAGAAG
Human-actin-R	GAAGGAAGGCTGGAAGAG
Mouse-Snail-F	GGTCCCCAACTACGGGAAAC
Mouse-Snail-R	GGTCCCCAACTACGGGAAAC
Mouse-Zeb1-F	GCTGGCAAGACAACGTGAAAG
Mouse-Zeb1-R	GCTGGCAAGACAACGTGAAAG
Mouse-Zeb2-F	ATTGCACATCAGACTTTGAGGAA
Mouse-Zeb2-R	ATAATGGCCGTGTCGCTTCG
Mouse-Twist1-F	GGACAAGCTGAGCAAGATTCA
Mouse-Twist1-R	CGGAGAAGGCGTAGCTGAG
Mouse-Twist2-F	CGCTACAGCAAGAAATCGAGC
Mouse-Twist2-R	CGGAGAAGGCGTAGCTGAG
Mouse-Slug-F	ACTGTATGGACATCGTCGGC
Mouse-Slug-R	ATGGGGGTCTGAAAGCTTGG
Mouse-N-Cadherin-F	GGGAGGGGTAAAAGTTCTTAGCA
Mouse-N-Cadherin-R	ATTCAGAACGCTGGGGTCAG
Mouse-E-Cadherin-F	GGACGTCCATGTGTGTGACT
Mouse-E-Cadherin-R	GATCAGAATCAGCAGGGCGA

Mouse-Vimentin-F	CGGCTGCGAGAGAAATTGC
Mouse-Vimentin-R	CCACTTTCCGTTCAAGGTCAAG

Supplementary Table 2. The primers have been used for ChIP-qPCR

Name	Sequence（5’-3’）
USP10 Promoter-F	AGGCAGGACTTGGGGAGTGAAT
USP10 Promoter-R	GAAGGGGAGAGGGCAGGCA

Supplementary Table 3. List of antibodies used in this study

Antibody	Source	Catalog
PLAGL2	GeneTex	GTX32095
USP10	GeneTex	D7A5
Vimentin	Proteintech	#10366-1-AP
Normal mouse IgG	Merck	12-371
Normal rabbit IgG	Merck	12-370
Ki67	Cell Signaling Technology	#9449
N-Cadherin	Proteintech	22018-1-AP
β-Actin	Proteintech	#66009-1-Ig
Anti-mouse IgG	Cell Signaling Technology	#14709
Anti-rabbit IgG	Cell Signaling Technology	#14708
GAPDH	Proteintech	60004-1-Ig
c-Myc	Proteintech	67447-1-Ig
ADRB2	Abcam	ab182136
Ubiquitin	Cell Signaling Technology	#3936

Supplementary Table 4. List of inhibitors used in this study

Inhibitor	Target	Source	Catalog
Propranolol	ADRBs	Selleck	S4076
Spautin-1	USP10	Selleck	S7888
ICI118551	ADRB2	Selleck	S8114
MG132	26s Proteasome	MedChemExpress	133407-82-6
Cycloheximide	Protein Synthesis	Coolaber	CC4071

Supplementary Table 5. The detailed information of HCC patients

ID	Gender	Age	Diagnosis	Stage	Microvascular invasion (MVI)
4888206	Male	61	HCC	Ⅱ	M1
4888923	Male	54	HCC	Ⅰ	M0
4889757	Female	62	HCC	Ⅰ	M0
4667795	Male	52	HCC	Ⅱ	M1
